# Vinorelbine and Intermittent Cyclophosphamide Sensitize an Aggressive Myc-Driven B-Cell Lymphoma to Anti-PD-1 by an Immunological Memory Effective against Tumor Re-Challenge

**DOI:** 10.3390/jcm12072535

**Published:** 2023-03-27

**Authors:** Stefania Orecchioni, Paolo Falvo, Giovanna Talarico, Giulia Mitola, Giulia Bravetti, Patrizia Mancuso, Paola Nicoli, Francesco Bertolini

**Affiliations:** 1 Laboratory of Hematology-Oncology, European Institute of Oncology IRCCS, Via Ripamonti 435, 20141 Milan, Italy; 2 Onco-Tech Lab, European Institute of Oncology IRCCS and Politecnico di Milano, 20141 Milan, Italy; 3 Department of Experimental Oncology, European Institute of Oncology IRCCS, Via Adamello 16, 20137 Milan, Italy

**Keywords:** lymphoma, cyclophosphamide, vinorelbine, metronomic chemotherapy, checkpoint inhibitors

## Abstract

We have previously shown in triple-negative breast cancer (TNBC) models that a triple therapy (TT) including intermittent cyclophosphamide (C), vinorelbine (V), and anti-PD-1 activates antigen-presenting cells (APC) and generates stem like-T cells able to control local and metastatic tumor progression. In the present manuscript, we report the generation of a highly aggressive, anti-PD-1 resistant model of a high-grade, Myc-driven B-cell non-Hodgkin’s lymphoma (NHL) that can be controlled in vivo by TT but not by other chemotherapeutic agents, including cytarabine (AraC), platinum (P), and doxorubicin (D). The immunological memory elicited in tumor-bearing mice by TT (but not by other treatments) can effectively control NHL re-challenge even at very high inoculum doses. TT re-shaped the landscape of circulating innate NK cells and adaptive immune cells, including B and T cells, and significantly reduced exhausted CD4^+^ and CD8^+^ TIM3^+^PD-1^+^ T cells in the spleens of treated mice.

## 1. Introduction

Immune checkpoint inhibitors (ICI) such as anti-CTLA-4, anti-PD-1, and anti-PD-L1 monoclonal antibodies have been successfully introduced in the therapy of a variety of cancer types [1]. However, at the present time, only a fraction of cancer patients benefit from ICIs, and in the large majority of cases, the clinical benefit is limited in time [2].

Cancer genomic and microenvironmental complexity suggests that in almost all patients only a combinatorial therapeutic approach can be successful [3]. For this reason, intense preclinical and clinical research is currently ongoing to define and validate in patients the best synergic strategy for ICI incorporation in cancer therapy.

We have described that a “two-hit” triple therapy (TT) [4,5], involving antigen-presenting cell (APC) activation by a vinca alkaloid such as vinorelbine (V) and the generation of new TCF1+ stem cell-like T cell (scT) clones by an intermittent dosage of the alkylating agent cyclophosphamide (C140), can significantly improve the efficacy of anti-PD-1 in two triple-negative breast cancer (TNBC) models otherwise poorly sensitive to ICIs. The TT effect was dependent upon C dosage and schedule, and due to T cells, as it was abrogated by the in vivo depletion of CD3^+^CD4^+^ and/or CD3^+^CD8^+^ cells [4,5,6].

To confirm that this approach can also be effective in different types of cancer and to investigate whether TT may generate an immunological anti-cancer memory, in the present study we describe a model of a very aggressive neoplasia resistant to anti-PD-1, i.e., a disseminated, orthotopic, Myc-driven B-cell lymphoma, and compared TT with other therapies currently used in the clinic for this disease. TT, but not other therapies, sensitized lymphoma cells to anti-PD-1, controlled lymphoma progression, and generated an immunological memory that in the large majority of mice avoided lymphoma generation even when lymphoma cells were re-injected in numbers significantly higher than in the previous inoculum.

## 2. Materials and Methods

### 2.1. Cell Lines

The LY27805 cell line was kindly provided by Bruno Amati’s group [7] at the European Institute of Oncology–Italian Foundation for Cancer Research (FIRC) Institute of Molecular Oncology (IEO–IFOM, Milan, Italy) campus, expanded and stored according to their instructions. Specifically, LY27805 cells were plated at 2 × 10^5^ cells/mL in B cell medium: 50:50 mixture of DMEM and IMDM (Euroclone, Pero (MI), Italy), 10% FBS (Euroclone, Pero (MI), Italy), 2 mM L-glutamine (Euroclone, Pero (MI), Italy), 1% penicillin/streptomycin (Euroclone, Pero (MI), Italy), 50 μM β-mercaptoethanol (Euroclone, Pero (MI), Italy), and non-essential amino acid (NEEA) (Euroclone, Pero (MI), Italy). Phoenix-Ampho cells were cultured with DMEM (Euclone, Pero (MI), Italy), 10%FBS (Euclone, Pero (MI), Italy), 2 mM L-glutamine (Euclone, Pero (MI), Italy), 1% penicillin/streptomycin (Euclone, Pero (MI), Italy).

Cells were tested and authenticated by the StemElite ID System (Promega, Madison, WI, USA). Cells were tested every six months for Mycoplasma by means of the ATCC Universal Mycoplasma Detection Kit 30-1012, cultured for no more than two weeks, and used for no longer than 15 passages.

### 2.2. Cell Line Infection

The LY27805 cell line was thoroughly transduced with retroviral vectors expressing stably luciferase under the control of a promoter: pRetro MI-Luciferase-IRES-mCherry (Addagene plasmid #75020). Phoenix-Ampho cells were co-transfected with 10 μg of retroviral vector, 6 μg of pKAT2 vector (helping retroviral particle-producing vector) using the Calcium Phosphate transfection system. After 16 h, media was removed and replaced with 5 mL of fresh medium to increase viral load.

Viral supernatant was then collected at 24 and 48 h and added directly to the LY27805 cell lines plated. In detail, viral supernatant was collected with a 0.22 μm sterile syringe filter and added directly to 2 × 10^5^ LY27805 cells supplemented with 8 μg/mL of polybrene (Sigma-Aldrich Merck, St. Louis, MO, USA). Spin Infection was performed for 1 h at 37 °C twice at 1800 rpm in a day for two separate days. Infection efficiency was then assessed using a fluorescence microscope (EVOS cells imaging system, Promega, Madison, WI, USA). Infected cells were then sorted using FACS Jazz (BD Bioscience, Franklin Lakes, NJ, USA).

### 2.3. In Vivo Experiments

Experiments involving animals were approved by the Italian Ministry of Health and have been performed in accordance with the applicable Italian laws (D.L. vo 26/14 and following amendments), the Institutional Animal Care and Use Committee, the institutional guidelines at the European Institute of Oncology, and the ARRIVE guidelines. In vivo studies were carried out in 8-week-old immune-competent C57BL/6J female mice (Envigo) in the animal facility at the IEO-IFOM campus.

To generate a syngeneic model of Non-Hodgkin lymphoma, 5 × 10^4^ to 2.5 × 10^5^ LY27805-RFP^+^Luc^+^ cells were injected intravenously (IV) in the tail vein of C57BL/6J mice. Tumor growth was monitored weekly using the In Vivo Imaging System (IVIS; PerkinElmer, Whaltam, MA, USA). Briefly, mice were intraperitoneally (IP) injected with 150 mg/kg of XenoLight D-Luciferin–K+ Salt Bioluminescent Substrate (PerkinElmer Whaltam, MA, USA). After 10 min, animals were anaesthetized with isofluorane apparatus and images acquired using Living Image Software (PerkinElmer Whaltam, MA, USA). Radiant efficiency was calculated on the basis of the epifluorescence signal, as indicated in the user manual. Treatment started about 7–10 days from tumor injection, when a bioluminescence signal was detectable in each individual mouse. Mice were observed daily throughout the treatment period for signs of morbidity/mortality.

### 2.4. In Vivo Therapy

C57BL/6J tumor-bearing mice (n = 5 per study arm) were treated with different drugs used as single agents or in combination. Drug dosages used in this study are the standard chemotherapeutic drugs for Non-Hodgkin lymphoma and doses were based on the literature data associated with no or acceptable toxicity, as well as no significant changes in mouse weight. Blood was collected at different time points from the tail vein, and circulating immune cells were determined by multiparametric, 10-colour flow cytometry. Cyclophosphamide was used at 140 mg/kg (C140) as already shown in Refs. [4,8,9]. Vinorelbine (V) was used at 9 mg/Kg as we have previously shown in Refs. [4,9]; Doxorubicin (D) was used at 2.9 mg/kg [4,10], Cisplatin (P) was used at 0.25 mg/kg [4,10], and Cytarabine (ara-C) was used at 15 mg/kg [11]. Chemotherapeutic drugs were dissolved in PBS and IP administrated once a week, every 6 days for 3 weeks. Mouse monoclonal anti-PD-1 targeting antibody was purchased from Bioxcell (Lebanon, NH, USA) (clone RMP1-14) and was administered IP 0.2 mg/mouse every 2 days for a total of 5 doses [4]. The therapeutic scheme is presented in Supplementary Figure S1.

### 2.5. Flow Cytometry

At least 100,000 cells per sample were acquired using a 3-laser, 10-color flow cytometer (Navios-Ex; Beckman Coulter, Brea, CA, USA) for T-cell receptor analysis or using a 13-color FACS Celesta (BD Bioscience) for immune cell population analyses. The antibodies list and gating strategies are summarized in the Supplementary Table S1. Lymphocytes and myeloid cells were characterized using state-of-the-art markers. Specifically, cells were gated for size, singlets, and then by positive and negative markers: CD3^+^CD4^+^ and CD3^+^CD8^+^ T cells, CD3-CD335^+^ NKs, CD19^+^ B cells, Gr1^−^CD11b^+^CD11c^+^ monocytes, SSC^high^CD11b^+^Gr1^+^ granulocytes, CD11c^+^CD11b^−^Gr1^−^ antigen-presenting cells (APC), SSC^low^CD11b^+^Gr1^+^ myeloid-derived suppressor cells (MDSC), and T cells were further analyzed to investigate the exhaustion status (Tim3^+^PD-1^+^).

### 2.6. T Cell Receptor Clonality

T-cell receptor (TCR) clonality was analyzed in the spleen using the FITC Hamster Anti-Mouse Beta TCR Kit (BD Biosciences, clone H57-597), following the manufacturer’s protocol. Briefly, the spleen was mechanically dissociated and 2 × 10^5^ cells were stained with an antibody cocktail mix containing: 7AAD (Beckman Coulter, Brea, CA, USA), CD45 PE-Cy7 (BD Biosciences, clone 30-F11), CD3 APC (BD Biosciences, clone 145-2C11), CD4 PE (BD Biosciences, clone GK1.5), CD8 APC-Cy7 (BD Biosciences, clone 53-6.7), and a specific region of VDJ beta chain rearrangement. Viable cells (7-AAD^−^) were gated for CD45/CD3/CD4 and CD45/CD3/CD8 positivity, and among them, we evaluated for each β chain rearrangement the percentage of positivity. Fold increase was determined by dividing the percentage of positive cells revealed in each experimental condition versus the untreated control. Finally, not engrafted mice were normalized to the engrafted ones.

### 2.7. Statistical Analysis

Data were expressed as means ± sem (in case of normal distribution). Normal distribution was assessed using the Shapiro Wilk’s normality test. To compare two sample groups, either the Student’s *t*-test or the Mann-Whitney U-test was used based on normal or not normal distribution. To test differences in survival, the Mantel-Cox test was performed. Statistical analysis was carried out with Prism 9.3.1. (GraphPad). 

## 3. Results

### 3.1. TT Sensitizes an Aggressive Myc-Driven B-Cell Lymphoma to Anti-PD-1 and Prevents Lymphoma Growth In Vivo

In our previous work [4,5], we demonstrated that TT, including C140, V, and anti-PD-1, eradicates tumor growth in two murine models of TNBC. We investigated whether the same TT combinatorial therapy can also be effective against a highly aggressive murine lymphoma model. To achieve this aim, we took advantage of a previous Myc-driven B-cell non-Hodgkin’s lymphoma cell line characterized by Bruno Amati’s laboratory [7].

**Figure 1:**
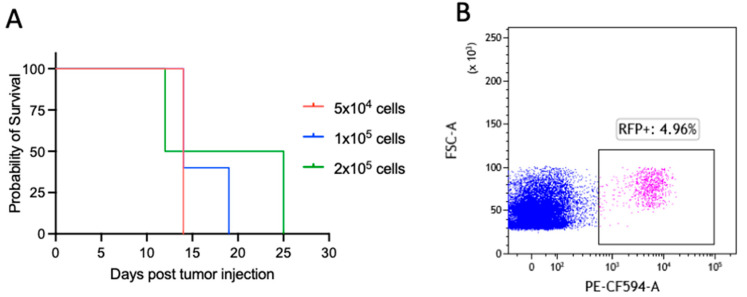
Ly27085-Luc is a lymphoma model with high penetrance and low latency. (**A**) Kaplan-Meier survival analysis of mice injected intravenously with 5 × 10^4^ (red line), 1 × 10^5^ (blue line), and 2 × 10^5^ (green line) Ly27805-Luc cells. The Mantel-Cox test was performed to assess statistical differences in survival between the three groups (n = 5 per experimental condition). (**B**) Representative flow cytometry plots showing percentage of RFP^+^ cells within the spleen of a control mouse. Cells were gated according to physical parameters.

These cells were retrovirally infected with pRetro MI-Luciferase-IRES-mCherry (Addagene plasmid #75020) plasmids carrying both an RFP gene (for in vitro selection) and a Luc gene (for in vivo tracking), herein called Ly27085-Luc. To characterize the latency and the penetrance of this lymphoma, mice were IV inoculated with three scalar concentrations of Ly27085-Luc cells (5 × 10^4^, 1 × 10^5^, and 2 × 10^5^) (Figure 1A). As shown, all mice died of lymphoma shortly after tumor inoculation (on average 20 days). Paradoxically, the higher number of cells did not correlate with higher aggressiveness, and with shorter latency. Moreover, taking advantage from the RFP selective marker, we assessed that very low percentages of lymphoma cells within the spleen were sufficient to kill the animal (Figure 1B). Taken together, these results indicate that Ly27805-Luc is an aggressive MYC-driven lymphoma model with very short latency (20 days on average) and high penetrance (100%).

**Figure 2:**
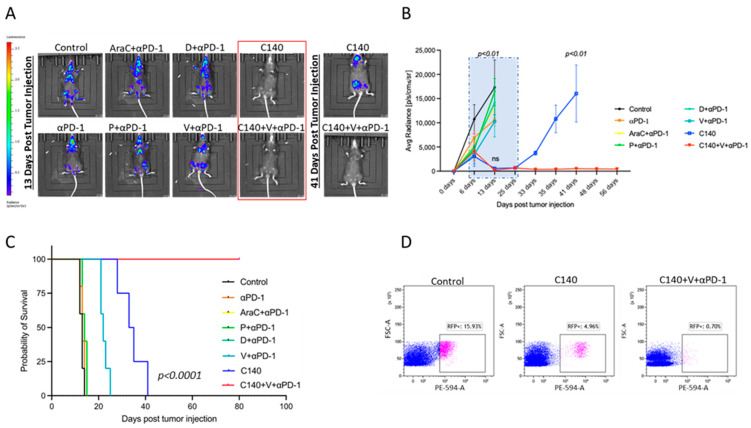
Ly27085-Luc lymphoma growth control by TT vs. other therapies. (**A**) In vivo live imaging of Ly27805-Luc tumors of mice treated with different experimental conditions. On the left is shown the luminescence/radiance bar. Red indicates a higher luminescence signal while blue is the lowest one. Mice were injected with 1 × 10^5^ Ly27805-Luc cells and monitored by IVIS. Images were taken 13 days after tumor injection. Mice in the red square did not show any difference, thus the mice were monitored for a longer period. On the right is shown the image 41 days after tumor injection. (**B**) Ly27085-Luc tumor growth curve of mice treated with chemotherapeutic agents, ICI, or chemotherapeutic agents combined with ICI. The dashed blue square represents the treatment period (from day 7 until day 28 post lymphoma injection). The Shapiro–Wilk test was performed to define normal distribution, and a 2-tail T Student’s test was applied. Statistical analysis was done for each condition compared with C140+V+anti-PD-1 (n = 5 per experimental condition). (**C**) Kaplan-Meier survival analysis of Ly27085-Luc-injected mice treated with chemotherapeutic agents, ICI, or chemotherapeutic agents combined with ICI. The Mantel-Cox test was performed to assess statistical differences in survival between the three groups (n = 5 per experimental condition). (**D**) Representative flow cytometry plots showing the percentage of RFP^+^ cells within the spleen of a control (left panel), C140- (middle) and C140+V+anti-PD-1-treated mouse. Cells were gated according to physical parameters.

To assess the efficacy of our TT approach [4,5], we injected C57BL/6J mice with 1 × 10^5^ Ly27805-Luc cells and treated them with several chemotherapeutic agents used in the clinic for B-cell lymphoma therapy, the anti-PD-1 ICI, and chemotherapeutic agents combined to ICI. We measured tumor growth by bioluminescence signals as shown in Figure 2A,B. In vivo studies indicated that intermittent (i.e., every 6 days, in a metronomic fashion) C140 was more effective than any other combinatorial regimens including ICI plus AraC, D, or P at their regular preclinical dosages. Both C140 alone and TT initially abrogated tumor growth, in line with our previous studies on TNBC [4,9]. Therefore, after interrupting the treatments, we prolonged the experimental observational time to more than 80 days and we found that the TT association with V and anti-PD-1 significantly increased the preclinical efficacy of C140 (*p* < 0.01). In addition, TT dramatically improved the overall survival rate compared with other combinatorial therapies (Figure 2C). All the C140-treated mice succumbed around 30–40 days after tumor injection, whereas the TT-treated mice were alive and without any signs of lymphoma (*p* < 0.0001) up to day 80, at the end of the observational study. At the end of the experiment, the animals were sacrificed, and the RFP signal was evaluated in the spleen, as shown in Figure 2D. In accordance with the luciferase signal, RFP-positive cells dramatically dropped after TT.

### 3.2. TT Reshapes the Intratumoral Immune Cell Landscape and Targets Terminally Exhausted T Cells

We and others have previously demonstrated that anti-PD-1 favors the selection of progenitor-exhausted T cells, with crucial effects over tumor growth [4,5,6]. Progenitor-exhausted T cells are defined to be Tcf1^+^ [12], whereas terminally exhausted T cells, which have poor anticancer function, are defined as PD-1^+^Tim3^+^ [13].

**Figure 3:**
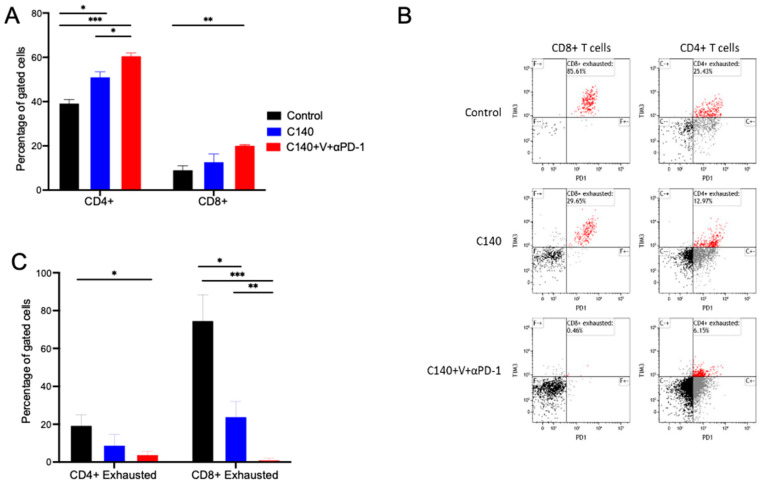
TT reshapes tumor infiltrating lymphocytes and reduces exhausted CD3^+^CD4^+^ and CD3^+^CD8^+^ T cells. (**A**) Percentage of CD3^+^CD4^+^ and CD3^+^CD8^+^ cells in the spleen of control (black), C140- (blue), and C140+V+anti-PD-1-treated mice (red). The Shapiro–Wilk test was performed to assess normal distribution, and thus 2-tails Student’s *t* test’ was applied. (* *p* < 0.05, ** *p* < 0.01, *** *p* < 0.001) (n = 5 per experimental condition). (**B**) Representative flow cytometry plots showing the percentage of exhausted CD3^+^CD8^+^ cells (defined as CD3^+^CD8^+^PD-1^+^TIM3^+^, on the left) and of exhausted CD3^+^CD4^+^ cells (defined as CD3^+^CD4^+^PD-1^+^TIM3^+^, on the right) within the spleen of a control (upper panel), C140- (middle), and C140+V+anti-PD-1-treated mouse (bottom panel). Cells were gated according to physical parameters. (**C**) Quantification of exhausted CD3^+^CD8^+^ cells (defined as CD3^+^CD8^+^PD-1^+^TIM3^+^) and of exhausted CD3^+^CD4^+^ cells (defined as CD3^+^CD4^+^PD-1^+^TIM3^+^) within the spleen of control, C140-treated, and C140+V+anti-PD-1-treated mice. The Shapiro–Wilk test was performed to define normal distribution, and thus 2-tails Student’s *t* test’ was applied (* *p* < 0.05, ** *p* < 0.01, *** *p* < 0.001) (n = 5 per experimental condition).

To determine whether anti-PD-1 might also have a similar function within our lymphoma model, we analyzed by flow cytometry the immune cell populations in the spleen of control, C140, and TT-treated mice. Animals (n = 5 per study arm) were inoculated IV with 1 × 10^5^ Ly27085-Luc cells and treated with C140 alone, TT (C140+V+anti-PD-1), or without any treatment. Control and C140-treated mice were sacrificed when moribund, TT-treated mice after 80 days, and the T cell composition within the spleen was analyzed (Figure 3).

As shown in Figure 3A, we observed that C140 alone and TT increase the percentage of CD3^+^CD4^+^ and CD3^+^CD8^+^ cells in the spleen. This increase is larger when one compares controls versus TT-treated mice (40% of CD3^+^CD4^+^ in the control versus 60% in the TT, *p* < 0.001 and 10% of CD3^+^CD8^+^ in the control versus 20% in the TT, *p* < 0.01). A statistically significant increase (*p* < 0.05) was observed also for CD3^+^CD4^+^ T cells between C140 and TT-treated mice.

We then analyzed terminally exhausted T cells, defined as CD3^+^CD4^+^/CD8^+^ PD-1^+^TIM3^+^ T cells. Surprisingly, we observed that terminally exhausted T cells were abundant in the control and decreased in the treated samples. This decrease was more profound in TT-treated mice. Specifically, we confirmed that TT was associated with a significant decrease in exhausted CD3^+^CD8^+^ T cells compared to controls (*p* < 0.001) and C140-treated mice (*p* < 0.01). A similar trend was also observed for exhausted CD3^+^CD4^+^ T cells (Figure 3B,C).

In line with results observed in TNBC models [4,5,6], these results in the lymphoma model suggest that TT reshapes the T cell landscape and selects for anti-tumor effective T cells.

### 3.3. TT Orchestrates the Activation of T Cell Antitumor Memory in Mice

**Figure 4:**
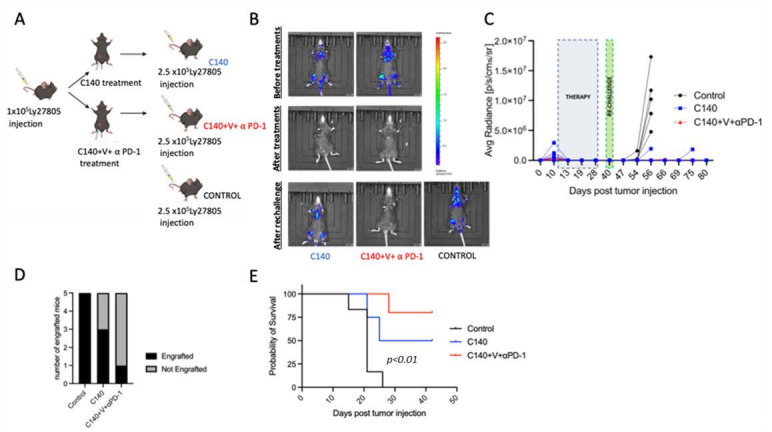
TT generates an anti-tumor immune memory. (**A**) Schematic representation of the tumor re-challenge experiment. Briefly, 10 C57BL/6J mice were injected IV with 1 × 10^5^ Ly27085-Luc cells; on day 10 post lymphoma injection, once systemic engraftment was confirmed, mice were randomized to receive different treatments (n = 5 per experimental condition) up to day 31; on day 40 post tumor injection, TT- and C140-treated mice were re-challenged with 2.5 × 10^5^ Ly27085-Luc cells (i.e., a 2.5-fold higher inoculum of lymphoma cells); in parallel, 5 C57BL/6J mice were injected with 2.5 × 10^5^ Ly27085-Luc cells as control. ). Graphic representation present was carried out with the Biorender software (https://app.biorender.com, accessed on 18 January 2023). (**B**) In vivo live imaging of Ly27805-Luc tumors in TT- and C140-treated and control mice. On the right is shown the luminescence/radiance bar. Red indicates a higher luminescence signal while blue is the lowest one. Images were taken before (upper panel), at the end of the treatment (middle panel), and after 2.5 × 10^5^ Ly27805-Luc lymphoma re-challenge (bottom panel). (**C**) Ly27085-Luc tumor growth of single re-challenged mice (black line control, blue line C140-cured and red line TT cured mice (n = 5 per experimental condition). The dashed blue square represents the treatment period (from day 10 until day 31 post lymphoma injection). The dashed green square represents Ly27805-Luc re-challenge on day 40 post-tumor injection. (**D**) Bar graph showing the number of lymphoma-engrafted mice (in black) over the number of not engrafted mice (in gray). (**E**) Kaplan-Meier analysis of re-challenged Ly27085-Luc mice. The Mantel-Cox test was performed to assess statistical differences in survival between the three groups (n = 5 per experimental condition).

As we previously demonstrated that T cells are the most likely mediators of the anti-tumor TT effect, we asked whether an immune cell memory was generated in TT-treated mice. To answer this question, we performed a tumor re-challenge experiment, as illustrated in Figure 4A. Briefly, mice were injected with 1 × 10^5^ Ly27805-Luc cells and the presence of lymphoma cells was assessed by IVIS analyses 10 days after tumor injection (Figure 4B, top panel). Mice were then randomized into two experimental groups, treated with C140 or TT. When C140 and TT were administered to lymphoma-bearing mice, tumors were completely eradicated after the first infusion (Figure 4B, middle panel). When we re-challenged TT- and C140-cured mice with a 2.5-fold higher number of lymphoma cells on day 9 after the end of the treatment (day 40 after tumor injection), all control (i.e., untreated) mice (5/5) developed signs of lymphoma about 10 days post tumor re-challenge. Three out of five C140-treated mice presented signals in the limbs with a growth kinetic similar to controls. Remarkably, only one out of five TT-treated mice presented lymphoma signs (Figure 4B, bottom panel, Figure 4 C,D). At the end of the study, all control mice, 3/5 C140-treated, and only 1/5 triple-treated mice died of lymphoma (*p* < 0.01) (Figure 4E).

These results suggest that TT efficiently generated some memory immune responses persisting after lymphoma eradication.

### 3.4. The Immune Cell Landscape in the Peripheral Blood Is Altered after Lymphoma Re-Challenge

**Figure 5:**
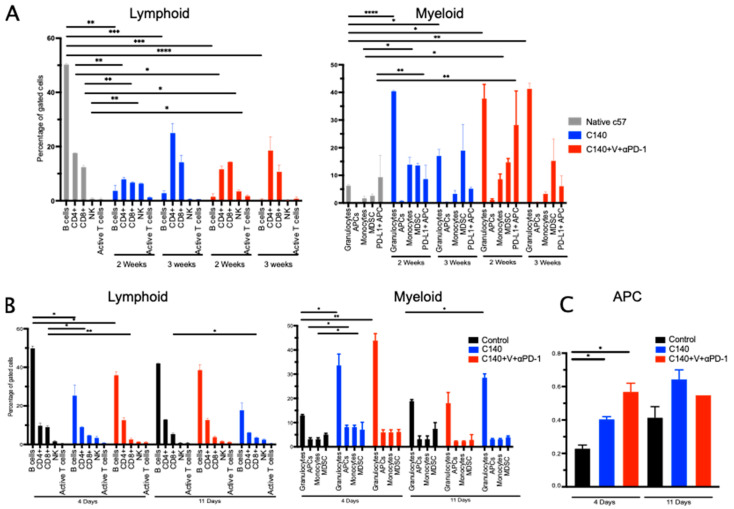
TT reshapes the immune cell landscape in the peripheral blood (PB). (**A**) Flow cytometry analysis in Ly27085-Luc bearing mice treated with C140 and TT at two different time points (two and three weeks after tumor injection). In the graph, major lymphoid (on the left panel) and myeloid cell subpopulations (on the right panel) are shown. Lymphoid comprises B cells (CD19^+^), CD3^+^CD4^+^ and CD3^+^CD8^+^ T cells, NK cells (CD335^+^), and activated CD8^+^T cells (CD3^+^CD8^+^CD69^+^CD25^+^). Myeloid cells include granulocytes (CD11b^+^Gr1^+^), APCs (CD11b^−^Gr1^−^D11c^+^), monocytes (CD11b^+^Gr1^−^CD11c^+^), myeloid-derived suppressor cells (MDSC) (CD11b^+^Gr1^+^), and PD-L1^+^APC (PD-L1^+^CD11b^−^Gr1^−^D11c^+^). The Shapiro-Wilk Test was applied to define normal distribution. A 2-tails Student’s *t* test’ was applied. Statistic is referred to each condition compared to untreated and not injected C57/BL6 mice (in grey in the graph). (* *p*  <  0.05, ** *p*  <  0.01, *** *p*  <  0.001, **** *p* < 0.0001). (**B**) Flow cytometry analysis in Ly27085-Luc re-challenged mice at two different time points (4 and 11 days after tumor re-challenge). In the graph major lymphoid (on the left panel) and myeloid cell subpopulations (on the right panel) are shown. Lymphoid comprises B cells (CD19^+^), CD3^+^CD4^+^ and CD3^+^CD8^+^ T cells, NK cells (CD335^+^), and activated CD8^+^T cells (CD3^+^CD8^+^CD69^+^CD25^+^). Myeloid cells include granulocytes (CD11b^+^Gr1^+^), APCs (CD11b^−^Gr1^−^D11c^+^), monocytes (CD11b^+^Gr1^−^CD11c^+^), myeloid-derived suppressor cells (MDSC) (CD11b^+^Gr1^+^), and PD-L1^+^APC (PD-L1^+^CD11b^−^Gr1^−^D11c^+^). The Shapiro-Wilk Test was applied to define normal distribution. A 2-tails Student’s *t* test’ was applied. Statistic is referred to each condition compared to untreated Ly27805-Luc injected C57/BL6 mice (in black in the graph). (* *p*  <  0.05, ** *p*  <  0.01). (**C**) Flow cytometry analysis in Ly27085-Luc re-challenged mice at two different time points (4 and 11 days after tumor re-challenge) for APC cells (defined as CD11b^−^Gr1^−^D11c^+^). The Shapiro-Wilk Test was applied to define normal distribution. A 2-tails Student’s *t* test’ was applied. Statistic is referred to each condition compared to untreated Ly27805-Luc injected C57/BL6 mice (in black in the graph). (* *p*  <  0.05).

As we have evidence that TT activates an immunological memory, we asked which types of immune cell subsets were altered during therapy and after the re-challenge with tumor cells. To this purpose, we analyzed by flow cytometry, at different time points, the percentages of circulating immune populations in the peripheral blood of untreated, TT- and C140-treated mice (Figure 5).

We first focused on the changes at two different time points (2 and 3 weeks) within the myeloid and lymphoid cell compartments after C140 or TT treatments (Figure 5A). As a control, we investigated five un-injected, untreated mice. We then focused on the same populations 4 and 11 days after the lymphoma re-challenge (Figure 5B,C).

B cells decreased after the treatments and increased after lymphoma re-challenge (*p* < 0.0001). This is consistent with our previous observations in TNBC and lymphoma models [4,9]. NK cells had a different kinetic, they initially increased during the TT (2 weeks) but were reduced in number after tumor re-challenge. We also analyzed the percentage of active T cells, defined as CD25^+^CD69^+^CD8^+^cells. Albeit not statistically significant, we observed an increase of active T cells after lymphoma re-challenge. These data deserve further molecular and phenotypic investigations, as they suggest an activation of T cells upon re-encounter with the antigens present on tumor cells.

Regarding the myeloid compartment, we did not observe any significant alterations in the peripheral blood for antigen-presenting cells (APC), monocytes, and myeloid-derived suppressor cells (MDSC). PD-L1^+^ APC significantly increased during the V-containing regimen (*p* < 0.01), while APC increased as previously described (Figure 5C) [4].

We observed that granulocyte numbers had a significant increase after treatment and after lymphoma re-challenge. We have observed a similar kinetic in another model of murine lymphoma [9], and these data deserve further investigation.

### 3.5. TT Decreases Terminally Exhausted T Cells upon Tumor Re-Challenge and Induces Specific T-Cell Clonal Selection

As already shown in Figure 1B and Figure 2D, lymphoma cells localize in the spleen. As we have not observed significant differences among adaptive immune cells in the peripheral blood of treated animals after tumor re-challenge, we focused our attention on splenic immune cell populations.

**Figure 6:**
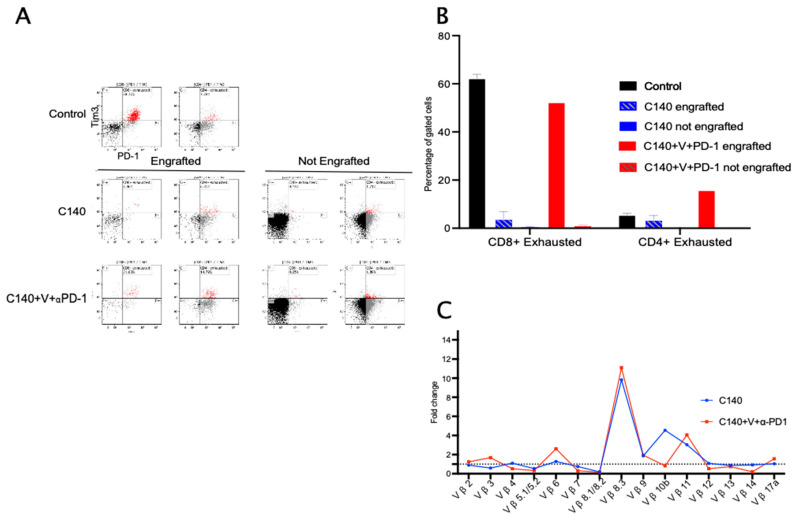
TT in tumor re-challenged mice selects for proliferative and oligoclonal T cells. (**A**) Representative flow cytometry plots showing the percentage of exhausted CD3^+^CD8^+^ cells (defined as CD3^+^CD8^+^PD-1^+^TIM3^+^, on the left) and of exhausted CD3^+^CD4^+^ cells (defined as CD3^+^CD4^+^PD-1^+^TIM3^+^, on the right) from the spleen of a control (upper panel), C140-engrafted (middle on the left), C140-not-engrafted (middle on the right), C140+V+anti-PD-1-engrafted (bottom on the left) C140+V+anti-PD-1-not engrafted (bottom on the right) mouse. Cells were gated according to physical parameters. (**B**) Quantification of exhausted CD3^+^CD8^+^ cells (defined as CD3^+^CD8^+^PD-1^+^TIM3^+^) and of exhausted CD3^+^CD4^+^ cells (defined as CD3^+^CD4^+^PD-1^+^TIM3^+^) from the spleen of a control (black), C140-engrafted (dashed blue), C140-not-engrafted (blue), and C140+V+anti-PD-1-engrafted (dashed red) and C140+V+anti-PD-1-not engrafted (red graph) mice. Two C140-treated mice did not engraft the lymphoma, one TT-treated mouse had lymphoma. (**C**) CD3^+^CD8^+^ TCR clonality in the spleen of lymphoma re-challenged mice. On the *x*-axis, 15 common Vβ chain recombinations are present, whereas on the *y*-axis the fold change increase is compared with engrafted mice (represented as dashed lines in the graph).

We analyzed by flow cytometry the percentage of exhausted CD3^+^CD4^+^ and CD3^+^CD8^+^ T cells along with PD-1^+^TIM3^+^ subpopulations in the spleen of lymphoma-engrafted and not engrafted mice. Exhausted T cells dramatically increased in the CD3^+^CD4^+^ and CD3^+^CD8^+^ subpopulations in lymphoma-bearing animals. At variance, in mice where lymphoma did not grow, we observed very few exhausted T cells (PD-1^+^TIM3^+^CD4^+^ were <2% and PD-1^+^TIM3^+^CD8^+^ were <1% of all gated cells). These data suggest a reduction of the suppressive immune response, in favor of an anti-tumoral activity. This evidence suggests that in the spleen, after lymphoma re-challenge, there is a selection for anti-tumor T cells (Figure 6A,B).

We then performed a T cell receptor (TCR) clonal analysis on CD3^+^CD8^+^ T cells collected from the spleen. We investigated, by flow cytometry, 15 common rearrangements of the TCR. In the case of a specific rearrangement, one evident peak is generated. As shown in Figure 6C, after tumor re-challenge we detected two specific peaks in mice resistant to lymphoma—corresponding to variants vβ8.3 and vβ11—when normalized over engrafted mice. These data indicate a switch towards oligoclonality in T cells.

Taken together, these data suggest that, in the tumor-infiltrated spleen, T cells might have a crucial role in the anti-tumor immunological memory elicitation, by inducing a shift towards more proliferative and anti-tumor T cells, and to an oligoclonal selection of T cells.

## 4. Discussion

ICI’s clinical impact on cancer therapy has been so far very relevant: in the past 10 years, eight immune checkpoint blockers against CTLA-4, PD-1, or PD-L1 have been approved for clinical use in more than 85 oncology indications [1,2]. However, at the present time, only a fraction of cancer patients benefit from ICI as a single therapy, and there is increasing evidence that, to improve ICI’s clinical efficacy, a combinatorial therapy is needed. The TT approach we have recently described in two TNBC models [4,5,6] includes drugs already in use in the oncology clinic for different indications and involves (a) APC activation by the vinca alkaloid vinorelbine and (b) the generation of new ScT clones by intermittent cyclophosphamide (i.e., every 6 days, in a metronomic fashion) [8] to overcome TNBC resistance to the anti-PD-1 ICI.

The kinetics of immune cell subsets during TT vs. other therapies have been described in Ref. [4]. Significant differences in the innate (myeloid and lymphoid) and adaptive subsets observed during these therapies might explain—at least in part—resistance to therapies other than TT and will be investigated in future studies.

V effects over APC activation have been previously described by us [9] and by the Takashima lab [14]. In the latter study, in a panel of 54 anti-cancer drugs, vinca alkaloids were ranked as the most prominent inducer of APC maturation; they increased CD40, CD80, CD86, and MHC II expression, triggered IL-1β, IL-6, and IL-12 p40 production, and augmented the capacity to activate T cells. The major hypothesis, still to be confirmed in mechanistic studies, is that partial and temporal disruption of intracellular microtubule networks by vinca alkaloids may be sensed by APCs as intrinsic danger signals.

The role of different dosages of the alkylating agent cyclophosphamide in improving ICI activity [15] and in generating an inflammatory neoplastic microenvironment that may foster CD3^+^CD4^+^ T-cell activity and an IFN/TNFalpha gene signature have also been recently described [16].

The present study shows that TT is also preclinically active in another type of otherwise ICI-resistant neoplastic disease, a high-grade, Myc-driven B-cell lymphoma. It should be noted that, in randomized clinical trials, TNBC patients seem to benefit from the association of ICIs with several chemotherapeutics including cyclophosphamide [17], whereas so far high-grade B-cell non-Hodgkin’s lymphoma patients have not been reported to receive a significant clinical benefit from ICIs, alone or in combinatorial therapies, apart from the case of mediastinal large-B cell lymphoma where the anti-PD-1 antibody is clinically active [18]. Thus, our data might be used to design future clinical trials in the non-Hodgkin’s lymphoma field. Notably, metronomic chemotherapy based on V and C has significant activity in patients with aggressive B-cell lymphomas, as long-lasting remissions were observed [19]. The tumoricidal activity of this combination may add to its immune-mediated effect, and this double effect may lay the groundwork for clinical translation.

In this context, Yu and colleagues [20] have recently suggested in single-cell studies that coinhibitory signals from the immune checkpoint molecules TIGIT and TIM-3 may drive T cell exhaustion in this disease. As clinically effective antibodies against TIGIT and TIM-3 are currently tested in trials, these ICI might deserve investigation in this neoplastic disease along with TT.

Another finding from the data reported here might be of interest for future clinical applications: we have found in the TNBC preclinical studies that TT efficacy was abrogated when CD3^+^CD4^+^ and/or CD3^+^CD8^+^ T cells were crippled in vivo by neutralizing monoclonal antibodies. Along a similar line, Zhang and colleagues [21] have recently identified subpopulations of CD3^+^CD4^+^CXCL13^+^ and CD3^+^CD8^+^CXCL13^+^ T cells that predict effective responses to ICI. In the present lymphoma model, we enlarge these observations with the finding that TT (and, to a significantly lesser extent, C140 alone) generate a preclinically active immunological memory against lymphoma re-challenge at doses 2.5 higher than the first lymphoma inoculum. We are now planning studies in TNBC and lymphoma models to pinpoint what immune cell subpopulation(s) is/are involved and crucial for this immunological memory. When phenotypically defined and purified, these cells might be transplanted for possible cellular therapies of TNBC, lymphoma, or other types of neoplastic diseases.

A possible caveat of the present study is related to differences in the immune systems of rodents and humans. We are now planning TT clinical trials that will be instrumental to confirm TT mechanisms of action in humans.

## Data Availability

Data are available upon reasonable request.

## Supplementary Materials

The following supporting information can be downloaded at: https://www.mdpi.com/article/10.3390/jcm12072535/s1. Table S1: Antibodies and gating strategies used in the study. Cell type is indicated in the first column, the phenotype in the second, fluorophores in the third, the clone and the distributors of the antibody in the fourth and fifth respectively. Figure S1: Therapeutic scheme used in this study.
