# Supplementary material for: Vinorelbine and Intermittent Cyclophosphamide Sensitize an Aggressive Myc-Driven B-Cell Lymphoma to Anti-PD-1 by an Immunological Memory Effective against Tumor Re-Challenge
Source: J Clin Med. 2023 Mar 27;12(7):2535. doi: 10.3390/jcm12072535 (PMC10095342; doi:10.3390/jcm12072535)

Supplementary Materials

Table S1.

Antibodies and gating strategies used in the study. Cell type is indicated in the first column, the phenotype in the second, fluorophores in the third, the clone and the distributors of the antibody in the fourth and fifth respectively.

Lymphocytes	Phenotype	Fluorophores	Clones	Catalogue number	Producer
T helper	CD3+CD4+	CD3 BV786	145-2C11	564379	BD Bioscience
		CD4 FITC	GK1.5	553729	BD Bioscience
T cytotoxic	CD3+CD8+	CD3 BV786	145-2C11	564379	BD Bioscience
		CD8 APC-Cy7	53-6.7	100714	BioLegend
NK	CD335+	CD335 Alexa-Fluor 700	29A1.4	561169	BD Bioscience
B cells	CD19+	CD19 APC	eBio1D3	17-0193-80	Invitrogen
Active T cytotoxic	CD3+CD8+CD25+CD69+	CD3 BV786	145-2C11	564379	BD Bioscience
		CD25 APC eFluor780	PC61.5	47-0251-82	Invitrogen
		CD69 BV605	H1.2F3	563290	BD Bioscience
		CD8 APC-Cy7	53-6.7	100714	BioLegend
**Myeloid cells**	**Phenotype**	**Fluorophores**	**Clones**	**Catalogue number**	**Producer**
Monocytes	Gr1-CD11b+CD11c+	Gr1 Pe-Cy7	RB6-8C5	25-5931-81	Invitrogen
		CD11b APC	M1-70	17-0112-82	Invitrogen
		CD11c FITC	HL3	557400	BD Bioscience
Granulocytes	SSC^{high}CD11b+Gr1+	Gr1 Pe-Cy7	RB6-8C5	25-5931-81	Invitrogen
		CD11b APC	M1-70	17-0112-82	Invitrogen
MDSC	SSC^{low}CD11b+Gr1+	Gr1 Pe-Cy7	RB6-8C5	25-5931-81	Invitrogen
		CD11b APC	M1-70	17-0112-82	Invitrogen
APC	CD11c+CD11b-Gr1-	Gr1 Pe-Cy7	RB6-8C5	25-5931-81	Invitrogen
		CD11b APC	M1-70	17-0112-82	Invitrogen
		CD11c FITC	HL3	557400	BD Bioscience
APCs PD-L1	CD11c+CD11b-Gr1-PD-L1+	Gr1 Pe-Cy7	RB6-8C5	25-5931-81	Invitrogen
		CD11b APC	M1-70	17-0112-82	Invitrogen
		CD11c FITC	HL3	557400	BD Bioscience
		PD-L1 PE	MIH5	558091	BD Bioscience
**Exhausted T cells**	**Phenotype**	**Fluorophores**	**Clones**	**Catalogue number**	**Producer**
T cytotoxic Exhausted	CD45+CD3+CD8+PD-1+TIM3+	CD3 BV786	145-2C11	564379	BD Bioscience
		CD4 FITC	GK1.5	553729	BD Bioscience
		CD8 APC-Cy7	53-6.7	100714	BioLegend
		PD-1 PE	RMP1-0	566831	BD Bioscience
		TIM3 BV421	5D12/TIM-3	747626	BD Bioscience
		CD45 PerCP	30-F11	557235	BD Bioscience
T Helper Exhausted	CD45+CD3+CD4+PD-1+TIM3+	CD3 BV786	145-2C11	564379	BD Bioscience
		CD4 FITC	GK1.5	553729	BD Bioscience
		CD8 APC-Cy7	53-6.7	100714	BioLegend
		PD-1 PE	RMP1-0	566831	BD Bioscience
		TIM3 BV421	5D12/TIM-3	747626	BD Bioscience
		CD45 PerCP	30-F11	557235	BD Bioscience

Supplementary Figure S1

Therapeutic scheme used in this study.

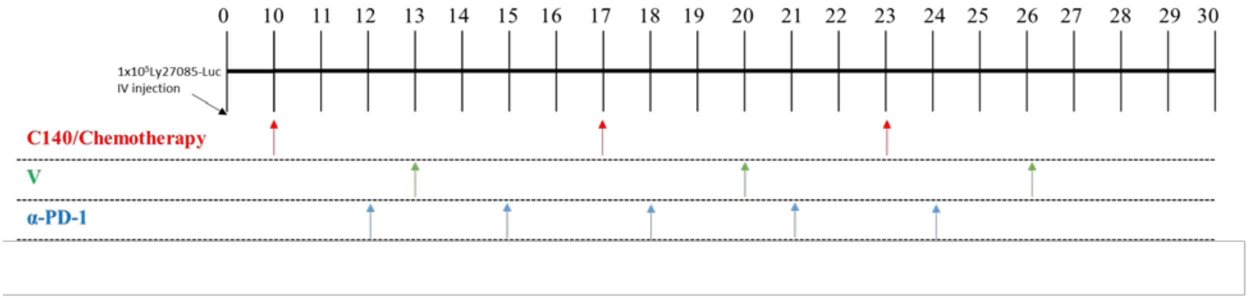

Supplement: Supplementary file 1 [file jcm-12-02535-s001.zip › jcm-2227171-supplementary.pdf]
